# The First Report of Anatomical Laparoscopic Liver Resection Performed Using ARTISENTIAL

**DOI:** 10.1111/ases.70172

**Published:** 2025-11-02

**Authors:** Go Shinke, Yutaka Takeda, Yoshifumi Iwagami, Mitsuru Kinoshita, Yoshiro Yukawa, Asami Arita, Kiminori Yanagisawa, Shinsuke Katsuyama, Masayuki Hiraki, Keijiro Sugimura, Masayoshi Yasui, Kohei Murata

**Affiliations:** ^1^ Department of Surgery Kansai Rosai Hospital Amagasaki Hyogo Japan

**Keywords:** ARTISENTIAL, hepatectomy, LLR

## Abstract

Laparoscopic liver resection (LLR) has been widely adopted; however, limited instrument mobility remains a challenge. Such technical limitations can be overcome with the novel articulating laparoscopic device ARTISENTIAL, but its use in LLR has not previously been described. Here we report the first anatomical liver resection performed using the ARTISENTIAL device. A 73‐year‐old man with a segment‐3 hepatic mass underwent laparoscopic left hepatectomy, with lymph node sampling. This procedure was performed using ARTISENTIAL. Parenchymal transection was performed using the clamp‐crushing technique with ARTISENTIAL. The Glissonian stump was closed with sutures. Compared to robotic systems, ARTISENTIAL provides similar motion benefits, without the associated cost or set‐up requirements. ARTISENTIAL is a versatile and cost‐effective technique. This case is the first reported anatomical LLR performed using ARTISENTIAL. Our experience suggests that this device can effectively overcome the major limitations of conventional instruments in LLR.

## 1 Introduction

Laparoscopic liver resection (LLR) has been increasingly adopted worldwide, with numerous reports highlighting its minimally invasive nature and safety [1, 2]. However, one of the main limitations of LLR is the restricted range of motion of conventional laparoscopic instruments. Although robot liver resection (RLR) has been proposed as a solution to this issue, several challenges remain, including high cost, limited access depending on institutional resources, and the fact that robotic devices are still under development [3].

ARTISENTIAL is a novel instrument designed to overcome the limited maneuverability of instruments in LLR. In 2019, LIVSMED Inc. (Seongnam, South Korea) launched ARTISENTIAL, a disposable, articulating, handheld laparoscopic instrument featuring multi‐degree‐of‐freedom dexterity and 360‐degree wrist‐like articulation at the end effector, similar to what is seen in robotic surgical systems [4]. The multi‐articulated function is achieved by the surgeon's wrist motion: horizontal wrist movement controls the distal end of the instrument tip, while vertical wrist movement controls the proximal end. The clinical application of ARTISENTIAL has been reported in various surgical fields [5, 6, 7, 8]. However, its use in highly invasive procedures such as complex hepatobiliary and pancreatic surgeries remains limited, and no prior reports have described its application in LLR [9]. Therefore, the utility of this device in such procedures remains unclear.

Here, we report the first anatomical liver resection performed using the ARTISENTIAL device and share our initial experience.

## 2 Case Presentation

**FIGURE 1:**
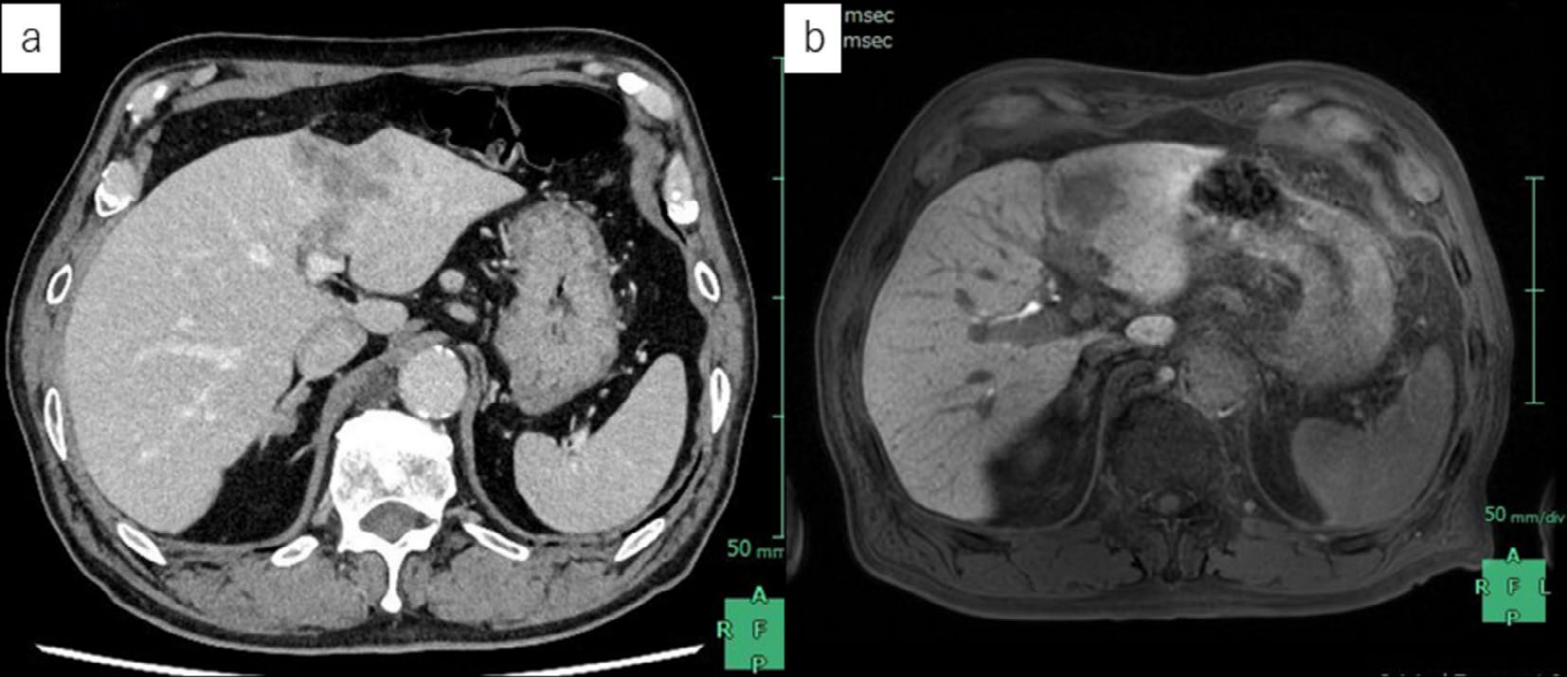
Preoperative imaging shows a 46‐mm tumor in liver Segment 3. (a) Contrast‐enhanced computed tomography. (b) Contrast‐enhanced magnetic resonance imaging.

**FIGURE 2:**
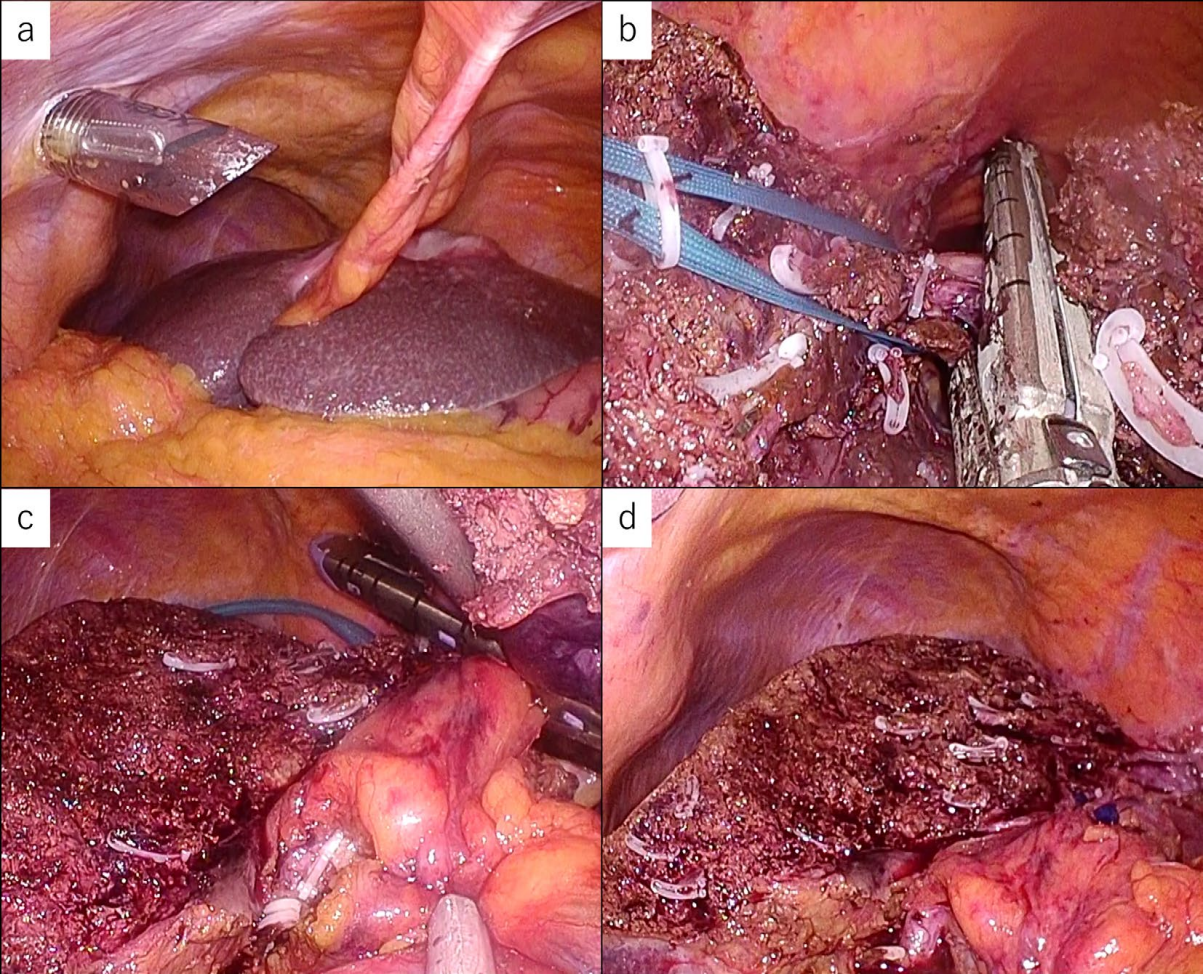
Intraoperative images. (a) The tumor previously identified in liver segment 3 is clearly visible. (b): The left hepatic vein was divided using a stapling device. (c): The left Glissonian pedicle was divided using a stapling device. (d): The transection surface after liver resection.

A 73‐year‐old man with a 46‐mm segment‐3 hepatic mass underwent laparoscopic left hepatectomy, with lymph node sampling (#8a and #12a) (Figures 1 and 2).

**FIGURE 3:**
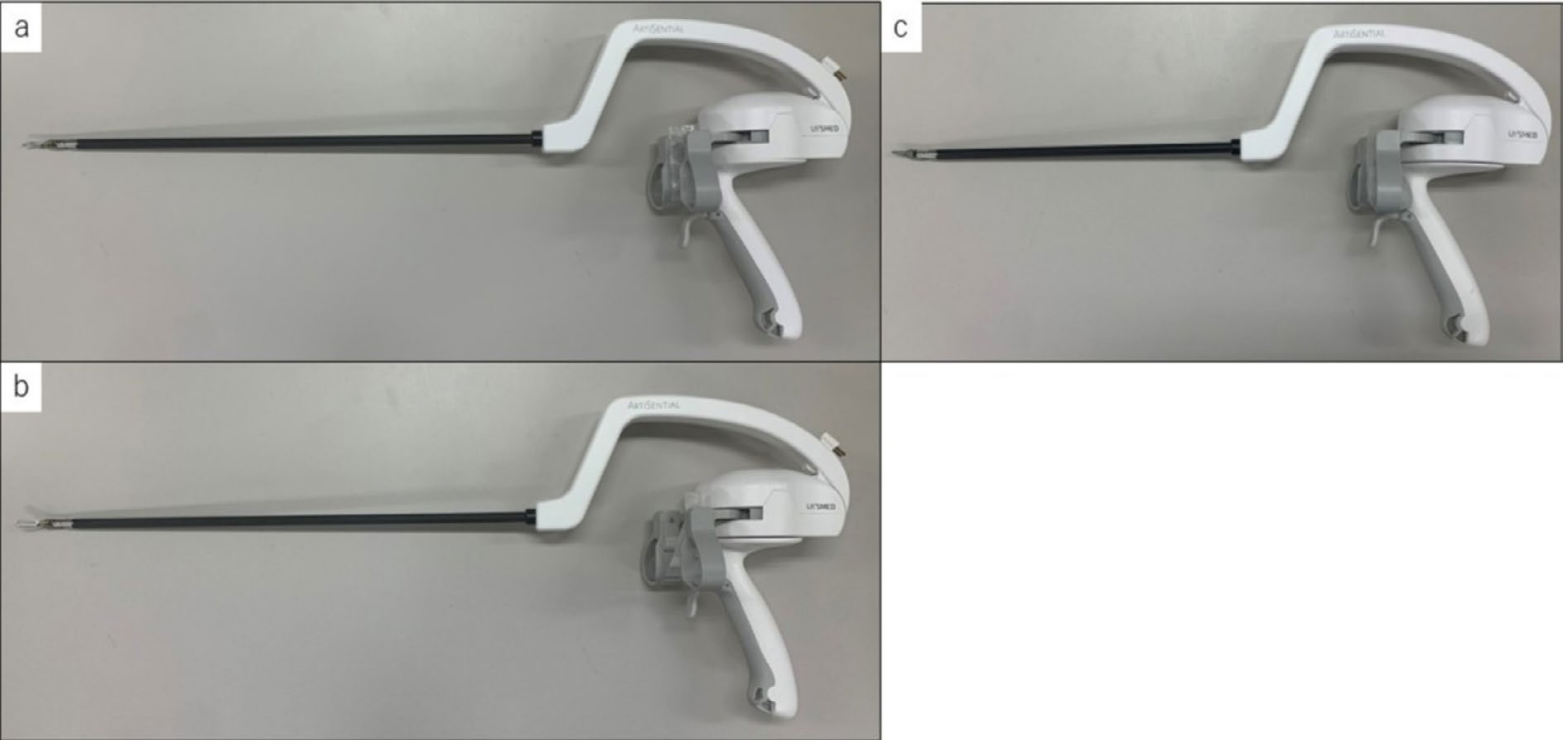
The complete exterior of the ARTISENTIAL instruments. (a) Bipolar precise dissector. (b) Bipolar fenestrated forceps. (c) Needle holder.

We used ARTISENTIAL instruments, specifically the Bipolar Precise Dissector, Bipolar Fenestrated Forceps, and Needle Holder (Figure 3). Parenchymal transection was performed using the clamp crushing technique with ARTISENTIAL. The Glissonian stump was closed with sutures (Video S1).

This study was conducted in accordance with the Declaration of Helsinki. This study was performed at Kansai Rosai Hospital and was approved by the local ethics committee (No. 2101014).

The operative time was 434 min. Blood loss was minimal. The patient was discharged on postoperative day 7, without complications. Final pathological evaluation revealed intrahepatic cholangiocarcinoma (pT4N1M0, Stage IVA) (based on the General Rules for the Clinical and Pathological Study of Primary Liver Cancer, 6th edition [revised version], Japan [10]).

## 3 Discussion

In LLR, limitations in the maneuverability of conventional instruments are often encountered in key surgical steps. Securing the main Glissonian pedicle is a critical step in anatomical LLR, but restricted instrument motion can make this technically challenging. Similarly, during parenchymal transection using the clamp crushing technique, the fixed directionality of instruments may hinder precision. Although suturing during liver transection is relatively uncommon, it can be extremely difficult depending on port placement and the suturing site, given the liver's limited mobility. As a result, metal clips are commonly used instead of sutures to reinforce the Glissonian stump. ARTISENTIAL effectively addressed these limitations, enhancing the surgeon's dexterity and control.

Second, when compared to robotic surgery, cost‐effectiveness is the most significant advantage of ARTISENTIAL. It frees institutions from the financial burden associated with the installation, maintenance, operation, and servicing of robotic systems. Using one ARTISENTIAL instrument in combination with conventional forceps may provide both cost reduction and sufficient benefits of ARTISENTIAL. Moreover, once the surgeon becomes proficient with ARTISENTIAL, it can be selectively used during conventional laparoscopic procedures when motion limitations arise.

Lastly, the use of ARTISENTIAL does require additional training. The operator underwent training using the manufacturer‐provided training kit before clinical use. This is likely due to the fact that ARTISENTIAL integrates the fulcrum mechanics of conventional laparoscopy with the intuitive, wrist‐like articulation commonly seen in robotic surgery—all within a single handheld device. Although we have not analyzed the learning curve in our own experience, previous reports have suggested that proficiency in ARTISENTIAL can be achieved after 19 cases of right hemicolectomy [6]. Accumulating more cases may allow us to further maximize the benefits of ARTISENTIAL.

## 4 Conclusion

This study represents the first report of an anatomical laparoscopic liver resection performed using ARTISENTIAL. Our experience suggests that this articulating laparoscopic device can effectively overcome the major limitations of conventional instruments in LLR.

## Author Contributions

G.S. and Y.T. designed the study, the main conceptual ideas, and the proof outline. Y.I. and M.K. collected the data. Y.Y., A.A., and K.Y. aided in interpreting the results and worked on the manuscript. K.S., M.Y. and K.M. supervised the project. G.S. wrote the manuscript with support from S.K. and Y.T. All authors discussed the results and commented on the manuscript.

## Conflicts of Interest

The authors declare no conflicts of interest.

## Supporting information


**Video S1:** This video demonstrates a laparoscopic left hepatectomy using ARTISENTIAL, highlighting liver mobilization, parenchymal transection, and suturing of the Glissonean stump.

## Data Availability

The data that support the findings of this study are available on request from the corresponding author. The data are not publicly available due to privacy or ethical restrictions.
